# Effect of Inpatient and Outpatient Pneumonia on Mobility Disability, Gait Speed, and Physical Activity in Older Adults

**DOI:** 10.3390/jcm10061236

**Published:** 2021-03-16

**Authors:** Joshua Brown, Reiko Sato, John E. Morley

**Affiliations:** 1 Rx-Epitome, LLC, Gainesville, FL 32606, USA; 2 Patient and Health Impact, Pfizer Inc., Collegeville, PA 19426, USA; reiko.sato@pfizer.com; 3 Division of Geriatric Medicine, Saint Louis University School of Medicine, St. Louis, MO 63104, USA; john.morley@health.slu.edu

**Keywords:** pneumonia, physical activity, physical functioning, older adults, gait speed, mobility

## Abstract

Pathophysiological changes caused by pneumonia may influence physical functioning in older adults. This study was a secondary analysis of the Lifestyle Interventions and Independence for Elders (LIFE) Study. The LIFE Study included 1635 individuals over an average follow-up of 2.6 years at eight clinical sites during 2010–2013. Adults ≥70 years-old with mobility limitations (Short Physical Performance Battery score ≤9) were randomized to a physical activity (exercise) intervention or health education control arm. This analysis evaluated the association between pneumonia events and major mobility disability (MMD), gait speed, and physical activity levels. Pneumonia events, classified as inpatient or outpatient, were assessed by self-report during longitudinal follow-up. MMD was measured by the inability to complete a 400-m walk test, or other proxies, as a binary outcome and separately analyzed as “short-term” and “long-term” MMD. Short-term MMD was defined as MMD occurring in the assessment period immediately following (between 1-day to 6-months after) a pneumonia event and long-term was in the following assessment period (6 to 12 months after the event). Short- and long-term gait speed was similarly recorded during the walk test in meters per second (m/s) and measured on a linear scale. Physical activity levels were captured via accelerometry and shown visually. Mixed-effects repeated measures regression adjusted for intervention assignment, baseline demographics, comorbid conditions, and frailty. Among the 1635 participants, *n* = 174 (10.7%) had a pneumonia event of which 80 (46% of events) were hospitalized. Those with pneumonia during follow-up had higher baseline medication use, prior hospitalizations, and higher prevalence of lung disorders but similar baseline functioning. Pneumonia hospitalization was associated with a 4-fold increase [OR = 4.1 (3.2–5.0)] and outpatient events were associated with a 2-fold increase [OR = 2.6 (2.1–3.1)] in the odds of short-term MMD. Pneumonia hospitalizations, but not outpatient events, were associated with a nearly 10% decrement in short-term gait speed. Pneumonia events were not associated with either long-term MMD or gait speed outcomes. Physical activity levels decreased from baseline immediately following the pneumonia episode (10–30% reductions) and returned to baseline after 6 months. These results emphasize the importance of managing pneumonia risk factors to prevent disease in order to maintain physical independence and activity in older adults.

## 1. Introduction

There is a significant risk of functional decline after precipitating health events, which can ultimately impact the quality of life and independence for older adults [1,2]. Functional disability in older adults is associated with increased mortality [3,4] and significantly increased healthcare utilization and expenditures [5] and thus has a significant societal impact in addition to its impact on patients and caregivers. The most serious precipitating events associated with disability can be characterized as severe events requiring inpatient care such as myocardial infarction, stroke, or serious falls and fractures. However, dependent on the vulnerability of a particular individual, more moderate events can also lead to disability [1].

Pneumonia is a common acute condition in older adults represented both by events managed in the outpatient setting as well as events requiring hospitalization. Pneumonia is an infection that can affect one or both lungs caused by bacteria, fungi, or viruses. Pneumonia is characterized by pulmonary infiltrates and inflammation in alveoli, which may lead to chest pain, difficulty breathing, and decreased blood oxygen levels [6,7,8]. An estimated 2 million episodes of pneumonia occur in older adults each year and about 40% of these will lead to hospitalization [6,7,8,9]. Pneumonia hospitalizations typically involve more severe, invasive disease or sepsis, and occur more frequently in older adults than other commonly considered conditions such as myocardial infarctions, stroke, or fractures [6,7]. Prior research has established a link between pneumonia hospitalization and the onset of physical and cognitive limitations [10,11,12]. In the Health and Retirement Survey, pneumonia hospitalization was associated with an average of one additional impairment in Activities and Instrumental Activities of Daily Living (I/ADLs) among those with no or mild-to-moderate baseline impairment and also had a lingering, long-term impact on these limitations [10].

While prior studies have shown a link between pneumonia hospitalizations and survey-reported functional disability measures such as I/ADLs, the current study aimed to evaluate the link between pneumonia events with objective measures of physical functioning and physical activity levels. This analysis specifically evaluated measures of mobility including the ability to walk 400-m and gait speed in the Lifestyle Interventions and Independence for Elders (LIFE) Study [13]. Different from prior studies, this analysis included pneumonia hospitalizations as well as pneumonia cases managed in a community setting (i.e., outpatient) and hypothesized that both inpatient and outpatient pneumonia events would be associated with decrements in physical functioning and activity levels in older adults.

## 2. Experimental Section

This is a secondary analysis of data collected from the LIFE Study. The LIFE Study was a multi-center, single-blind, parallel randomized trial conducted across eight centers in the United States between February 2010 and December 2013 [13]. The study protocol was approved by the institutional review boards of each institution [13]. Written informed consent was obtained from all study participants. The trial was monitored by a data and safety monitoring board appointed by the National Institute on Aging. The LIFE Study was registered with www.clinicaltrials.gov (accessed on 22 February 2010) prior to participant enrollment in the trial (NCT01072500). LIFE Study data for this study was provided by the National Institute on Aging’s AgingResearchBiobank after ethical approval by an independent review board.

Details of the study design, rationale, and characteristics of the full study population are described elsewhere [13,14]. Participants were eligible for the trial who were 70–89 years of age, scored <10 on the Short Physical Performance Battery (SPPB), were sedentary with ≤125 min of activity per week, and were able to complete the 400-m walk test within 15 min without sitting, leaning or without assistance.

### 2.1. Intervention

Details of the study interventions were previously published [13,15]. The physical activity (PA) intervention involved walking, with a goal of 150 min per week, strength, flexibility, and balance training. The intervention included attendance at two center-based visits per week and home-based activity three to four times per week for the duration of the study. The PA sessions were individualized and progressed toward a goal of 30 min of walking daily at moderate intensity, 10 min of primarily lower-extremity strength training by means of ankle weights (2 sets of 10 repetitions), 10 min of balance training, and large muscle group flexibility exercises.

The health education (HE) intervention included weekly educational workshops during the first 26 weeks, and then monthly sessions thereafter. Workshops included topics relevant to older adults, such as how to effectively negotiate the health care system, how to travel safely, preventive services and screenings recommended at different ages, where to go for reliable health information, nutrition, etc. The workshops did not include any PA topics. The program also included a 5- to 10-min instructor-led program of gentle upper extremity stretching or flexibility exercises.

### 2.2. Follow-Up Visits and Outcome Assessment

Details of MMD ascertainment were reported previously [16]. Briefly, participants were asked to walk 400 m at their usual pace, and MMD was defined as the inability to complete the walk within 15 min without sitting and without the help of another person or walker. When MMD could not be objectively measured because of the inability of the participant to come to the clinic and absence of a suitable walking course at the participant’s home, institution, or hospital; an alternative adjudication of the outcome was based on objective inability to walk 4 m in less than 10 s, or self-, proxy-, or medical record-reported inability to walk across a room. If participants met these alternative criteria, they were considered to be unable to complete the 400-m walk within 15 min. Gait speed was measured based on completion time of the 400-m walk test or from the SPPB. Participants were assessed every 6 months at clinic visits. Home, telephone, and proxy assessments were attempted if participants could return to the clinic. The assessment staff were masked to the intervention assignment and remained separate from the intervention team. Participants were asked not to disclose their assigned intervention arm or talk about their interventions during the assessment.

Accelerometry data (steps and metabolic cost [“METs”] measures) were captured in consecutive time periods at baseline, 6 months, and 12 months using a hip-worn device [13]. Participants in the LIFE Study were asked to wear the devices at all times except during sleep, bathing, or swimming for a 7-day period [17]. These measures were corrected to account for non-wear time and taken as total daily averages and as total daily averages during moderate-intensity activities defined as activities with an overall cadence of 760 counts per minute (captured along the vertical axis in 1-s epochs) [17]. The measurement periods limited the analysis only to those with pneumonia events at the 6-month assessment period and are compared to those without events by plots and relative percent changes.

### 2.3. Intervening Health Events

Participants or their proxies self-reported all healthcare utilization and reasons for that utilization since the last clinical visit. For hospitalizations, medical records were abstracted for diagnoses and procedures. Medical record assessors were blinded to the intervention assignment and Medical Officers at each clinical site used standardized criteria (Medical Dictionary for Regulatory Activities, MedDRA) to categorized reasons for hospitalization. Pneumonia managed in the community setting was captured by the self-report with a directed question related to “have you been diagnosed with pneumonia.” There was no further adjudication of outpatient pneumonia cases beyond self-report [13].

### 2.4. Statistical Analyses

Cohort demographics and baseline characteristics at baseline were described between those with and without a pneumonia event during the LIFE Study.

A repeated measures framework for each LIFE Study participant was used and included all follow-up assessments recorded. Pneumonia exposures were recorded with dummy variables for inpatient or outpatient pneumonia events. MMD and gait speed were analyzed using two measures which included short-term and long-term assessments. Short-term measures were those that were captured in the assessment period that would have immediately followed an event, i.e., the assessment occurred between 1-day and up to 6-months after the pneumonia event. Long-term measures were captured in the following assessment period, i.e., measured between 6-months and up to 12-months after the pneumonia event. MMD was recorded as a binary outcome and analyzed via repeated-measures logistic regression with fixed effects for baseline variables and random effects within individuals. Gait speed was analyzed as a continuous linear outcome with similarly mixed effects. These models were adjusted for intervention assignment, baseline demographics, functioning assessments, and other intervening health events including other hospitalizations. Baseline covariates modeled as fixed effects included intervention assignment, age (per year), sex, SPPB ≤ 7 or >7, race (White, Black, Other), education (≥high school), smoking status (current, former, never), and medical history (cardiovascular disease, diabetes, arthritis, lung disease, heart attack), self-rated health, and quintiles of an internally derived frailty index [18]. Odds ratios and 95% confidence intervals were reported from the MMD analysis and the adjusted percent change reported from the gait speed analysis. Accelerometry data were graphed in simple linear plots and not further analyzed given the small sample size. All analyses were conducted in SAS Enterprise Guide v8.1 with an alpha = 0.05 for all analyses.

## 3. Results

Among the 1635 participants randomized during the study period, there was an average of 2.6 years of follow-up time with a loss to follow-up of 4% annually. Over the course of this follow-up, *n* = 174 (10.7%) had a pneumonia event of which *n* = 80 (46% of all pneumonia events) were hospitalized. Those with pneumonia events during follow-up were mostly similar to the overall LIFE Study cohort but had a higher number of medications (average of 5.9 vs. 4.8; *p* < 0.001), higher prevalence of prior hospitalizations (14.4% vs. 7.5%; *p* = 0.002), and had an overall higher prevalence of lung-related disorders (e.g., pneumonia and bronchitis). There was similar respiratory function between the two groups measured by forced expiratory volume after 1 s (FEV1) and maximum inspiratory pressure as well as baseline physical functioning measures such as gait speed and SPPB score (Table 1).

**Figure 1:**
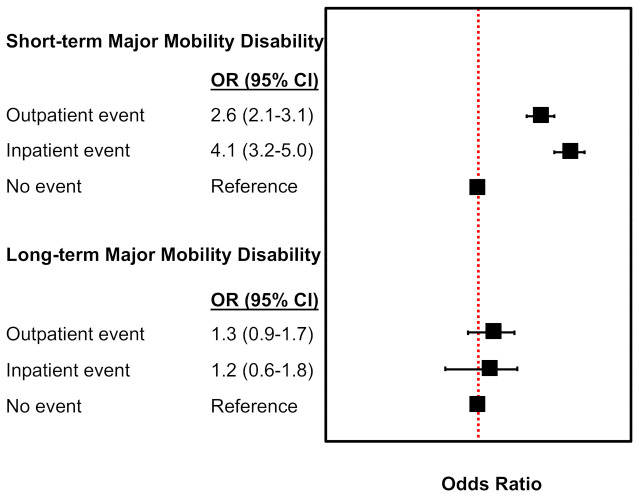
Adjusted odds ratios (OR) and 95% confidence intervals (95% CI) for the association be Table 1. or the null value.

Pneumonia events not requiring hospitalization were associated with more than double [OR = 2.6 (2.1–3.1)] the odds of experiencing short-term MMD during the LIFE Study compared to those without any pneumonia events (Figure 1). Similarly, those with pneumonia requiring hospitalization were at more than 4-times [OR = 4.1 (3.2–5.0)] higher odds of short-term MMD during follow-up compared to those without pneumonia events. Pneumonia events were not associated with long-term MMD (Figure 1).

**Figure 2:**
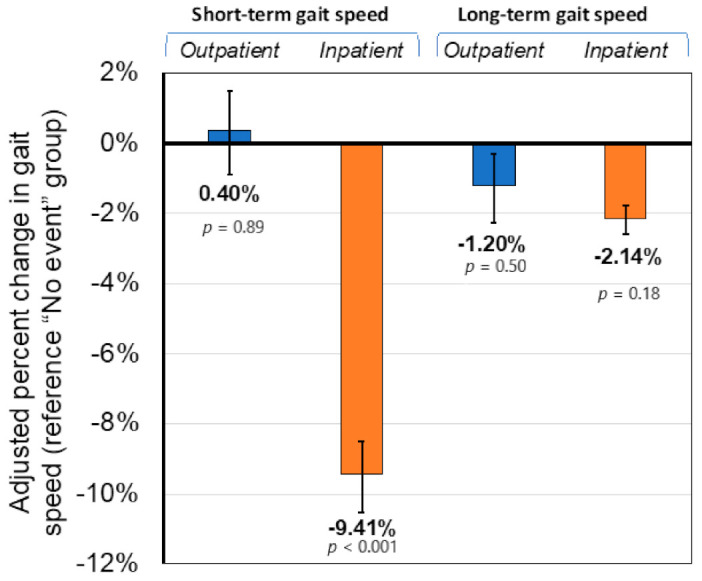
Short- and long-term adjusted percent change in gait speed after an inpatient or outpatient pneumonia event. Labels indicate the point estimate and the *p*-value for the comparison to the “No event” group.

For gait speed (Figure 2), pneumonia hospitalization was associated with a 9.1% (8.3–10.2%; *p* < 0.001) short-term decrease in gait speed while non-hospitalized pneumonia was not significantly associated with changes in short-term gait speed (0.3% (−1.1–1.5%); *p* > 0.05). Pneumonia was associated with small (1–2%) decreases in gait speed long-term, but these estimates were not statistically significant.

**Figure 3:**
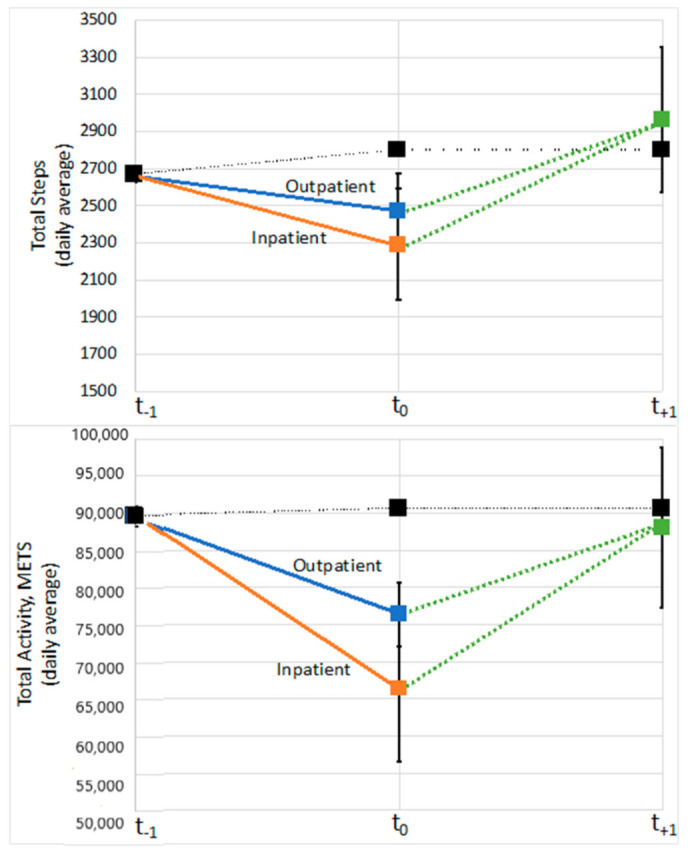
Change in total steps and total metabolic cost (METs) from the period prior, during, and immediately after a pneumonia episode. Group means were similar at baseline and those with outpatient (blue) and inpatient (orange) pneumonia events decreased then returned to baseline in the subsequent period (green). Those without any pneumonia events are shown in black. Due to sample sizes, inpatient and outpatient pneumonia groups were grouped together in the post-period.

**Figure 4:**
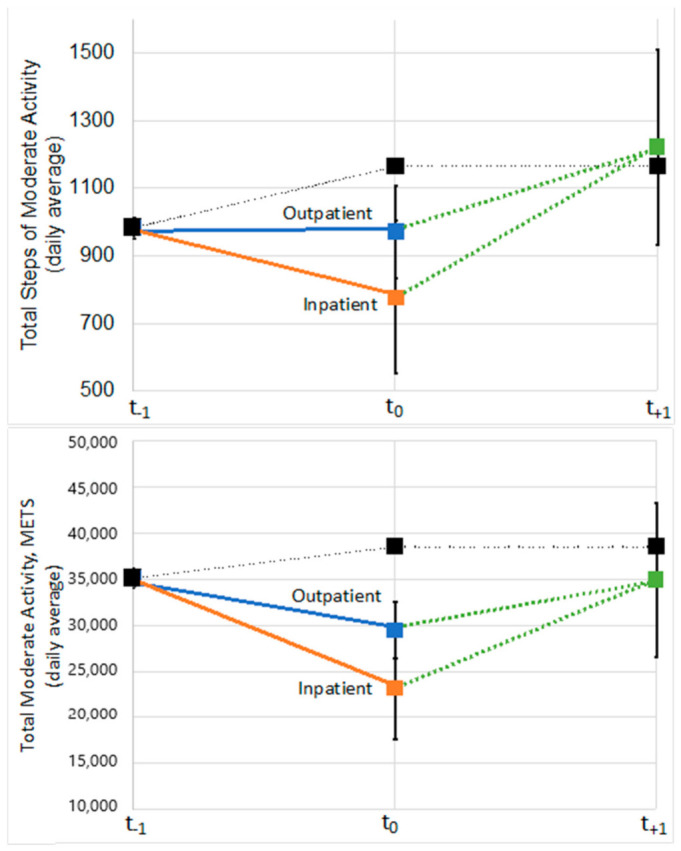
Change in total steps during moderate-intensity activities and total metabolic cost (METs) during moderate-intensity activities from the period prior, during, and immediately after a pneumonia episode. Group means were similar at baseline and those with outpatient (blue) and inpatient (orange) pneumonia events, decreased, then returned to baseline in the subsequent period (green). Those without any pneumonia events are shown in black. Due to sample sizes, inpatient and outpatient pneumonia groups were grouped together in the post-period.

Baseline (t_−1_) group step and activity levels were similar for the no pneumonia, inpatient pneumonia, and outpatient pneumonia in total steps and METs as well as total steps and METs during moderate activity (Figure 3 and Figure 4). At t_0_, which represents the assessment period immediately following a pneumonia event, total steps were 11.6% and 18.3% lower for those with outpatient and inpatient pneumonia episodes compared to those with none (Figure 3). Similarly, total METs were 15.7% and 26.7% lower for outpatient and inpatient pneumonia episodes compared to those without. For total steps and METs during moderate-intensity activities, levels were decreased for those with outpatient (16.7% and 23.3%) and inpatient (33.4% and 39.4%) episodes compared to the no pneumonia group, respectively (Figure 4). For each measure, the groups with pneumonia episodes returned to similar activity levels in the t_+1_ period and were grouped due to small sample sizes based on the measurement periods of accelerometry data in the LIFE Study.

## 4. Discussion

This study evaluated the association between both inpatient and outpatient pneumonia events and physical functioning and physical activity levels. There was a significant association between inpatient and outpatient pneumonia on short-term MMD but no long-term effects were noted. Inpatient pneumonia but not outpatient pneumonia was associated with significant decreases in gait speed in the short- but not long-term periods. Accelerometry showed that pneumonia episodes were associated with decreases in activity levels, which returned to normal within six months of the event.

These findings demonstrate the impact of pneumonia events on physical functioning—specifically mobility. Unique to this study, these results showed that pneumonia events not resulting in hospitalization had more than twice the odds of short-term MMD. This risk was even higher, on the order of 4-fold higher odds compared to no event, if the pneumonia event involved a hospitalization. Pneumonia hospitalizations were also associated with an approximate 10% reduction in short-term gait speed, but no reductions were observed for non-hospitalized pneumonia. For both outcomes of MMD and gait speed, pneumonia was not associated with long-term changes. Activity levels for those with pneumonia events dropped during the acute phase but were shown to return to similar levels as those without pneumonia events during the subsequent 6-month follow-up.

The effect of hospitalizations on physical functioning has been previously explored. In an analysis of the LIFE Study [19], all-cause hospitalizations were associated with 3-fold increased rates of MMD and suggested that recovery from MMD was lessened, though that finding was not statistically significant. Further, that study established that there was not an interaction between the LIFE Study intervention assignment, hospitalization, and MMD nor modification of this risk based on baseline SPPB scores [19]. That LIFE Study analysis, however, did not separate out cause-specific hospitalizations, including no specification for pneumonia-related events. In the aforementioned Health and Retirement Survey study which did account for pneumonia hospitalizations [10], the outcomes assessed are not comparable but generally show that pneumonia hospitalizations can impact varying domains of physical functioning. Different in that study compared to the current is the observation of a long-term effect, which may be a result of the different outcomes measured [10]. The current study also adds an assessment of outpatient pneumonia events, which were associated with short-term MMD and changes in physical activity levels but not long-term effects.

Pneumonia is among the most common acute conditions in older adults and a top cause of morbidity and mortality [6,8,20]. The link between pneumonia and reduced physical functioning is not well understood but is likely to include pathophysiological changes in lung function and may also be multi-factorial. For example, increased inflammation during and after pneumonia episodes is well documented and may be associated with reduced exercise capacity, sarcopenia, and additional health events such as myocardial infarction and stroke [21,22,23]. Our results suggest that these changes and their associated impact are acute as we observed no long-term decrements in mobility after the initial events. How pneumonia impacts the ability of older adults to live and function independently is of growing interest as the risk of pneumonia is modifiable with risk factor management and vaccination [20]. The stronger observed effect for hospitalization events was expected but also underscores the importance of specifically preventing severe pneumonia.

Pneumonia is associated with common risk factors such as chronic lung diseases, smoking, and heavy alcohol use. Other chronic and acute conditions as well as medications that suppress the immune system also increase the risk of pneumonia including malignancy, chronic renal failure, sickle cell disease, asplenia and immunosuppressant use. Management and modification of these risk factors may reduce the risk and severity of pneumonia [20]. The Centers for Disease Control recommends vaccines to further reduce the risk of pneumonia, including Influenza vaccination and pneumococcal vaccination [24]. However, both pneumococcal influenza vaccination rates continue to be subpar in older adults [25,26,27] even though vaccines are considered one of the most underutilized, high-value primary care practices available [28].

### Limitations

This study is strengthened by a clinically relevant cohort of older adults and frequent objective measures of mobility outcomes. There was potential for recall bias for outpatient pneumonia events as these relied on self-report compared to adjudicated records for hospitalized pneumonia events. This may have biased the results to the null as it is more likely that outpatient events are underreported. In addition, while the cohort was selected based on the LIFE Study criteria, this may limit the generalizability of these results to the overall older adult population and calls for additional research in more generalizable cohorts. Pneumonia events were ascertained “since the last follow-up” but were not more detailed in timing. Thus, events could occur between 1-day to 6-months prior to short-term mobility assessments and between 6–12 months for long-term assessments, which would make the results more conservative as the acute effects could wane the further an assessment was from the pneumonia event.

## 5. Conclusions

In conclusion, there was increased mobility disability as well as reductions in gait speed and physical activity levels in the acute (<6 months) period after pneumonia hospitalizations in an older adult cohort from the LIFE Study. These measures returned to baseline after 6 months. Outpatient pneumonia episodes were associated with mobility disability but not reductions in gait speed or activity levels. These results underscore the importance of preventing serious pneumonia episodes among older adults by encouraging lifestyle modifications and risk factor management to maintain mobility and independence as a way to support healthy aging.

## Figures and Tables

**Table 1:** Baseline demographic and characteristic comparison between those with and without a pneumonia event during follow-up.

	No Pneumonia (*n* = 1461)		Pneumonia (*n* = 174)		
	*n*/mean	%/SD	*n*/mean	%/SD	*p*-Value
Demographics					
Age, years	78.84	5.18	79.22	5.65	0.367
Female	992	67.9%	114	65.5%	0.526
Race					0.146
Black	262	17.9%	21	12.1%	
Other	94	6.4%	13	7.5%	
White	1086	74.3%	138	79.3%	
Education ≥ high school	985	67.4%	121	69.5%	0.572
Smoking					0.379
Former	647	44.3%	86	49.4%	
Current	44	3.0%	6	3.4%	
Number of medications	4.84	3.09	5.86	3.49	<0.001
Hospitalization, baseline	109	7.5%	25	14.4%	0.002
Assigned to physical activity intervention arm	735	50.3%	98	56.3%	0.134
Self-rated health					0.155
Excellent	101	6.9%	8	4.6%	
Very good	414	28.3%	48	27.6%	
Good	715	48.9%	78	44.8%	
Fair	214	14.6%	37	21.3%	
Poor	17	1.2%	3	1.7%	
Comorbidities/Medical History					
Heart attack	145	9.9%	17	9.8%	0.949
Heart failure	86	5.9%	14	8.1%	0.261
Stroke	103	7.0%	17	9.8%	0.193
Cancer	345	23.6%	47	27.2%	0.321
Diabetes	402	27.5%	50	28.9%	0.734
Arthritis	284	19.4%	38	22.0%	0.452
Respiratory Functioning					
Pneumonia	479	32.8%	82	47.1%	<0.001
Bronchitis	534	36.6%	77	44.3%	0.047
Cough	192	13.1%	37	21.3%	0.004
Phlegm	209	14.3%	29	16.7%	0.404
Chronic bronchitis	107	7.3%	22	12.6%	0.014
Emphysema	47	3.2%	12	6.9%	0.014
Asthma	202	13.8%	36	20.7%	0.015
Inhaler use	126	8.6%	33	19.0%	<0.001
Forced Expiratory Volume after 1 s (FEV1), L	1.9	0.6	1.8	0.6	0.015
Maximum inspiratory pressure	58.9	22.6	59.9	22.5	0.604
Physical functioning tests					
SPPB ≤ 7	650	44.5%	90	51.7%	0.070
SPPB	7.4	1.6	7.2	1.7	0.142
CHAMPS score	16.6	32.5	21.0	36.8	0.098
PAT-D Disability Domain 1: Basic ADL Score	1.3	0.4	1.4	0.4	0.329
PAT-D Disability Domain 3: Instrumental ADL	1.1	0.3	1.1	0.3	0.643
400-m Walk gait speed (m/s)	0.82	0.17	0.8	0.16	0.141
Self efficacy rating: 400 m walk	74.3	21.38	72.32	19.53	0.246
Grip strength	24.07	9.78	23.84	10.93	0.773
HRQL Stressed Score	11.12	6.05	11.07	6.3	0.919
3MSE: Total Score (max = 100)	91.68	5.41	90.89	5.44	0.070
Deficit accumulation frailty index score	0.26	0.06	0.27	0.06	0.200

Abbreviations: SPPB = Short Physical Performance Batter; CHAMPS = Community Health Activities Model Program for Seniors; PAT-D = Pepper Assessment for Disability; ADL = Activities of Daily Living; HRQL = Health-Related Quality of Life; 3MSE = Modified Mini Mental State Examination; SD = Standard Deviation.

## Data Availability

Data are available upon request.
